# QTL analysis of domestication syndrome in zombi pea (*Vigna vexillata*), an underutilized legume crop

**DOI:** 10.1371/journal.pone.0200116

**Published:** 2018-12-18

**Authors:** Sujinna Dachapak, Norihiko Tomooka, Prakit Somta, Ken Naito, Akito Kaga, Peerasak Srinives

**Affiliations:** 1 Department of Agronomy, Faculty of Agriculture at Kamphaeng Saen, Kasetsart University, Nakhon Pathom, Thailand; 2 Genetic Resources Center, Gene bank, National Agriculture and Food Research Organization, Kanondai, Tsukuba, Ibaraki, Japan; 3 Soybean and Field Crop Applied Genomics Research Unit, Institute of Crop Science, National Agriculture and Food Research Organization, Kanondai, Tsukuba, Ibaraki, Japan; Università Politecnica delle Marche, ITALY

## Abstract

Zombi pea (*Vigna vexillata* (L.) A. Rich) is an underutilized crop belonging to the genus *Vigna*. Two domesticated forms of zombi pea are cultivated as crop plants; seed and tuber forms. The cultivated seed form is present in Africa, while the cultivated tuber form is present in a very limited part of Asia. Genetics of domestication have been investigated in most of cultivated *Vigna* crops by means of quantitative trait locus (QTL) mapping. In this study, we investigated genetics of domestication in zombi pea by QTL analysis using an F_2_ population of 139 plants derived from a cross between cultivated tuber form of *V*. *vexillata* (JP235863) and wild *V*. *vexillata* (AusTRCF66514). A linkage map with 11 linkage groups (LGs) was constructed from this F_2_ population using 145 SSR, 117 RAD-seq and 2 morphological markers. Many highly segregation distorted markers were found on LGs 5, 6, 7, 8, 10 and 11. Most of the distorted markers were clustered together and all the markers on LG8 were highly distorted markers. Comparing this *V*. *vexillata* linkage map with linkage maps of other four *Vigna* species demonstrated several genome rearrangements in *V*. *vexillata*. QTL analysis for 22 domestication-related traits was investigated by inclusive composite interval mapping in which 37 QTLs were identified for 18 traits; no QTL was detected for 4 traits. Number of QTLs detected in each trait ranged from 1 to 5 with an average of only 2.3. Five QTLs for tuber width and three QTLs for tuber weight. Interestingly, 2 QTLs each for tuber width and tuber weight detected on LG2 and LG4 were located at similar position and wild allele increased tuber width and weight. This indicated wild germplasm having small tuber have potential to increase yield of large tuber cultivated type. Large-effect QTLs (PVE > 20%) were on LG4 (pod length), LG5 (leaf size and seed thickness), and LG7 (for seed-related traits). Comparison of domestication-related QTLs of the zombi pea with those of cowpea (*Vigna unguiculata*), azuki bean (*Vigna angularis*), mungbean (*Vigna radiata*) and rice bean (*Vigna umbellata*) revealed that there was conservation of some QTLs for seed size, pod size and leaf size between zombi pea and cowpea and that QTLs associated with seed size (weight, length, width and thickness) in each species were clustered on same linkage.

## Introduction

The genus *Vigna* is an important taxon of leguminous plants. This genus comprises more than 100 species that distribute in all major continents including Africa, America, Asia, Australia and Europe. Among those species, as high as nine *Vigna* species are domesticated/cultivated. These domesticated *Vigna* species include azuki bean (*Vigna angularis* (Wild.) Ohwi and Ohashi), black gram (*Vigna mungo* (L.) Hepper), créole bean (*Vigna reflexo-pilosa* Hayata), mungbean (*Vigna radiata* (L.) Wilczek), moth bean (*Vigna aconitifolia* (Jacq.) Maréchal), rice bean (*Vigna umbellata* (Thunb.) Ohwi and Ohashi), cowpea (*Vigna unguiculata* (L.) Walps.), Bambara groundnut (*Vigna subterranea* Verdc.) and zombi pea (*Vigna vexillata* (L.) A. Rich) [1,2]. The former six species are of Asian origin and belong to the same subgenus *Ceratotropis*, while the latter three species are of African origin. Cowpea and Bambara groundnut belong to the subgenus *Vigna*, while zombi pea belongs to the subgenus *Plectrotropis*. These crops are generally cultivated by resource-poor farmers as a sole crop or a component in various cropping systems.

Among the domesticated *Vigna* species, zombi pea is the least known crop. Two forms of zombi pea exists; seed type and tuber (storage root) types. The seed type is believed to be domesticated in Sudan (Africa) [3,4], while the tuber type is believed to be domesticated in Bali and Timor, Indonesia (Asia) [5] and India [6,7]. Genetic diversity analysis in a large set of *V*. *vexillata* germplasm using simple sequence repeat (SSR; also known as microsatellite) markers revealed clear difference between the two types and suggested that these two types were domesticated from different wild gene pools of *V*. *vexillata* [4]. The seed type zombi pea is classified as variety *macrosperma*. Possibly, this type is domesticated from wild zombi pea of East Africa [4]. It has erect growth habit and large seed size, and is insensitive to day length and no seed dormancy with some degree of pod dehiscence. The variety *macrosperma* is highly cross-compatible with wild *V*. *vexillata* [8]. The tuber type zombi pea has not yet been classified as a variety of *V*. *vexillata*. It has viny growth habit and large seed size, and is sensitive to day length and no seed dormancy with some degree of pod shattering. The tuber type is grown principally for fresh edible tuber roots which contain as high as 15% of proteins [5,6]. This cultivated type has been reported to be cross-incompatible with wild *V*. *vexillata* and the variety *macrosperma* [8]. The phenotypic difference between the two cultivated types of *V*. *vexillata* is a result of divergent selection during the evolution of the crop. Morphological and physiological differences that occur during the change from wild plants to cultivated crops is known as “domestication syndrome” [9]. Crop domestication is an accelerated evolutionary process that is the result from human intentional and unintentional selection and natural selections [10]. Domestication-related traits in legume crops include plant architecture (determinate growth habit), gigantism in the consumed plant organs (seed size and/or pod size), reduced seed dispersal (indehiscent pod), loss of seed dormancy and no response to photoperiod [11–13].

Knowledge on genetic basis of crop domestication is useful for identification of beneficial gene(s) in the wild relatives of the cultivated crops that can be used in plant breeding. Moreover, such knowledge can also provide insight into crop evolution and agriculture, such that the case of genetic analysis of indehiscent pod in soybean by Funastuki [14] which showed crucial role of the trait in the global expansion of soybean cultivation. Generally, genes controlling crop domestication are identified by quantitative trait loci (QTL) analysis. Among crops of the genus *Vigna*, QTL mappings of domestication syndrome traits have been carried out in azuki bean [13,15], mungbean [16], rice bean [17], cowpea/yardlong bean [18–21]. The genetics of domestication of zombi pea is of particular interest because two cultivated forms of this legume have experienced different domestications from wild zombi pea. Seed type zombi pea was domesticated from wild zombi pea of Africa, while tuber type zombi pea was domesticated from wild zombi pea of Asia [4,22]. Information on the genetics of domestication-related traits would be useful for zombi pea improvement programs, and for comparative genome studies among members of the genus *Vigna*. The objectives of this study were to identify QTLs for domestication-related traits in tuber type zombi pea and to compare them with previously reported QTLs for domestication in other *Vigna* species.

## Materials and methods

**Fig 1:**
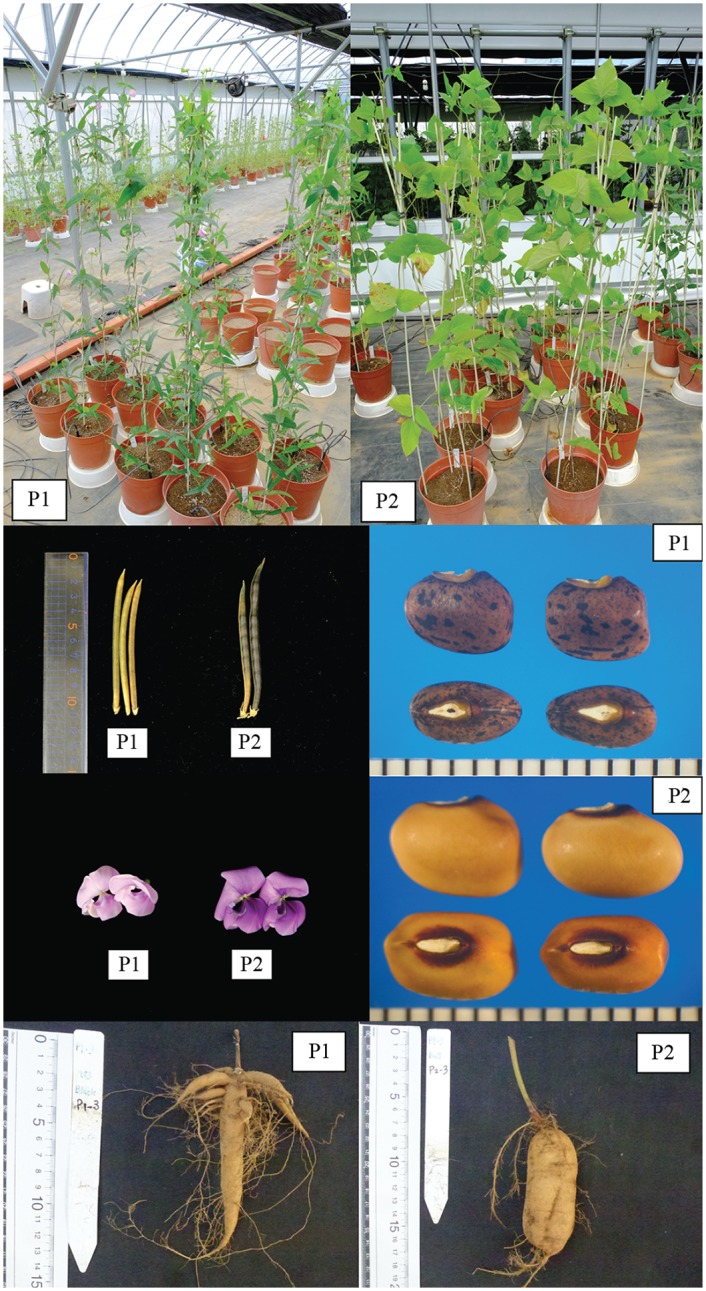
Comparison of morphological characteristics between wild *V*. *vexillata* accession “AusTRCF66514” and cultivated *V*. *vexillata* accession “JP235863” used in this study.

An F_2_ population of 139 plants was used in this study. The population was developed from a cross between accession JP235863 and accession AusTRCF66514 (Fig 1). JP235863 is a cultivated zombi pea collected from Bali Island, Indonesia which it is cultivated principally for edible tuber root, while AusTRCF66514 is a wild zombi pea from India. AusTRCF66514 was crossed with JP235863 as female and male parents, respectively, to produce F_1_ hybrid. Only one F_1_ seed was obtained and was self-pollinated to generate F_2_ population. One hundred and eighty seven F_2_ seeds together with parental seeds were sown in pots (1 seed per 1 pot) during June to November 2015 under a greenhouse condition at National Agriculture and Food Research Organization, Tsukuba, Japan. One hundred and fifty eight seeds germinated and developed into plants, but 139 of them grew vigorously and set flowers, and were used for DNA analysis and phenotypic data collection.

### DNA extraction

Total genomic DNA was extracted from young leaves of the parental and the F_2_ plants using a CTAB method described by Lodhi et al. [23]. DNA quality and quantity was checked by comparing with a known concentration ʎ-DNA (50 and 100 ng/μl) on 1% agarose gel. DNA concentration of each plant was adjusted to 5 ng/μl for PCR analysis. The adjusted DNA was also checked for quality using NanoDrop 8000 (Thermo Fisher Scientific, Wilmington, U.S.A.).

### Trait measurements

Twenty-two traits related to domestication in *Vigna* crops [13,15–17,20] were measured/evaluated (Table 1). Among those traits, seed color (SDC) and presence of black mottles (SDCBM) on seeds were considered as qualitative trait, while the others were considered as quantitative trait. Seedling traits, primary leaf width (LFPW) and primary leaf length (LFPL) were recorded when the first trifoliate was appeared. Stem thickness (STT), 10^th^ node length (STL10), maximum leaf length (LFML) and maximum leaf width (LFMW) were collected when eleventh trifoliate leaf was fully developed. Seed-related traits including number of seeds per pod (SDNPPD) was an average from ten pods, seed width (SDW), seed length (SDL) and seed thickness (SDT) was the average from ten seeds while 100-seed weight (SD100WT) was recorded from 100 seeds of each plant. The number of days from planting to first flowering (FLD) was recorded. Number of days to first mature pod (PDDM) chosen from first flower that had been developed to mature pod. After harvesting all pods, ten pods from each plant were used to measure/recorded for pod width (PDW), pod length (PDL), pod dehiscence (PDT) or number of twist along the length of pod (NTWP). PDT was recorded after the pods were kept in hot air oven at 40°C for 24 hours. Tuber traits (NTB, TBWT, TBW and TBL) were collected at 260 days after planting. Broad-sense heritability of each traits was calculated by using the following equation; ${\text{h}}^{2}=(\sigma_{F2}^{2}-(\sigma_{P1}^{2}-\;\sigma_{P2}^{2})/2)/\sigma_{P}^{2}$, where h^2^ is broad-sense heritability, $\sigma_{F2}^{2}$ is the variance of F_2_ population, $\sigma_{P1}^{2}$ is the variance of JP235863 and $\sigma_{P2}^{2}$ is the variance of AusTRCF66514.

**Table 1:** Domestication-related traits determined in F_2_ population derived from cross between cultivated (Bali) and wild (India) accessions.

General attribute	Organ	Trait	Trait abbreviation	QTL/gene	Evaluation
**Pod dehiscence**	Pod	Number of twists (count)	PDT	*Pdt*	Number of twists along the length of shattered pod
**Gigantism**	Seed	100-seed weight	SD100WT	*Sd100wt*	Weight of 100 seeds
		Length (mm)	SDL	*Sdl*	Maximum distance from top to bottom of the seed
		Width (mm)	SDW	*Sdw*	Maximum distance from hilum to its opposite side
		Thickness (mm)	SDT	*Sdt*	Maximum distance between both side of the hilum
	Pod	Length (mm)	PDL	*Pdl*	Length of straight pod
		Width (mm)	PDW	*Pdw*	Maximum width
	Stem	Thickness (mm)	STT	*Stt*	Stem diameter under the primary leaf (measured at flowering stage)
	Leaf	Primary leaf length (mm)	LFPL	*Lfpl*	Distance from pulvinus to leaf tip
		Primary leaf width (mm)	LFPW	*Lfpw*	Maximum width
		Maximum leaflet length (mm)	LFML	*Lfml*	Length of the largest terminal leaflet on leaves between node on first trifoliate leaf and node on ten trifoliate leaf
		Maximum leaflet width (mm)	LFMW	*Lfmw*	Width of the largest terminal leaflet on leaves between node on first trifoliate leaf and node on tenth trifoliate leaf
	Tuber	Number of tuber	NTB	*Ntb*	Number of storage root
		Tuber width	TBW	*Tbw*	Maximum width of main tuber
		Tuber length	TBL	*Tbl*	Distance from top of tuber to bottom of main tuber
		Tuber weight	TBWT	*Tbwt*	Weight of tubers
**Plant type**	Stem	Length (up to 10^th^ node) (cm)	STL10	*Stl10*	Length from node on primary leaf to node 10 of trifoliate leaf
**Earliness**	Flower	Days to first flower (day)	FLD	*Fld*	Number of days from planting to 1^st^ flowering
	Pod	Days to maturity of 1^st^ pod (day)	PDDM	*Pddm*	Number of days from 1^st^flowering to harvesting of 1^st^ pod
**Yield potential**	Seed	Number of seeds per pod (seeds/pod)	SDNPPD	*Sdnppd*	Number of seed per pod
**Pigmentation**	Seed	Seed coat color	SDC	*Sdc*	Brown (wild) or yellow (cultivated)
		Black mottle on seed coat	SDCBM	*Sdcbm*	Present (wild) or absent (cultivated)

### SSR marker analysis

A total of 1,876 SSR primer pairs (827 pairs from azuki bean [24,25], 562 pairs from cowpea [19,26], 318 pairs from mungbean [27–29], and 169 pairs from common bean [30–32]) were screened for polymorphism between the wild and cultivated parents. PCR amplification and fragment analysis for the primers from azuki bean, cowpea, and common bean were performed following the method described by Marubodee et al. [33], while for the primers from mungbean were the same as described by Somta et al. [34]

### RAD-seq analysis

RAD-seq analysis was conducted following a modified protocol described by Peterson et al. [35]. Genomic DNA of parents and each F_2_ plant was digested by double-digest RAD library method. In brief, 10 ng of genomic DNA was digested with EcoRI and BgIII (New England Biolabs, Ipswichs, MA, USA), and purified. Each adaptor with unique 4–8 bp index was ligated to digested DNA samples. The adaptor sequence was as follows: TruSeq_EcoRI_adaptor 1 = A*A*TTGAGATCGGAAGAGCACACGTCTGAACTCCAGTC*A*C TruSeq_EcoRI_adaptor 2 = G*T*CAAGTTTCACAGCTCTTCCGATC*T*C (* = variable index sequences to identify the individual DNA sample). BglII adaptor = A*A*TGATACGGCGACCACCGAGATCTACACTCTTTCCCTACACGACGCTCTT*C*CTruSeq_BglII_adaptor 2 = G*A*TCGGAAGAGCTGTGCAGA*C*T. The adaptors were ligated at 37°C overnight in 10 μl volume which contained 1 μl of 10x NEB buffer 2, 0.1 μl of 100x BSA (New England Biolabs), 0.4 μl of 5 uM EcoRI adapter and BgIII adapter, 0.1 μl of 100 mM ATP and 0.5 μl of T4 DNA ligase (Enzymatic, Beverly, MA, USA). The reaction solution was purified with AMPure XP (Beckham Coulter, CA, USA). Three microliter of the purified DNA was used in PCR amplification in 10 μl volume, containing 1μl of each 10 μM index and TruSeq universal primer, 0.3 μl of KOD-Plus-Neo enzyme and 1 μl of 10x PCR buffer (TOYOBO, Osaka, Japan), 0.6 μl of 25 mM MgSO_4_ and 1 μl of 10 mM dNTP. Thermal cycling was initiated with 94°C step for 2 mins, followed by 20 cycles of 98°C for 10 s, 65°C for 30 s and 68°C for 30 s. The PCR products were pooled and purified again with AMPure XP. The purified DNA was then loaded to a 2.0% agarose gel and fragments with size of about 320 bp were retrieved using E-Gel SizeSelect (Life Technologies, Carlsbad, CA, USA), the library was sequenced with 51 bp single-end read in one lane of HiSeq 2000 (Illumine, San Diego, CA, USA).

### Extraction of RAD-tag and bi-allelic RAD-marker detection

RAD-tag sequences were extracted and bi-allelic RAD-markers were detected using Stacks ver. 1.30 [36] following the procedures described by Marubodee et al. [33]. With the Stacks pipeline, the low quality sequences were filtered to obtain 51 bp RAD-tag sequence reads, and classified the RAD-tags sequences into the parental accessions and F_2_ individuals. Then ran denovo_map.pl program where RAD-tags with less than two sequence mismatches were grouped as a stack, parental stacks with less than three mismatches were estimated as those derived from homologous loci and lists of RAD-tag sequences and their count were constructed for each sample. We called genotypes of F_2_ individuals only when stacks have more than five RAD-tags (minimum depth of 5).

### Linkage map construction using SSR ad RAD-seq markers

A genetic linkage map of the F_2_ population was constructed using software Join Map 4.0 [37]. Polymorphic markers showing more than 10% missing genotypic data were excluded from linkage analysis. Chi-square analysis was used to test segregation of the marker loci (1:2:1 for co-dominant markers and 3:1 for dominant markers). The markers were grouped using minimum logarithm of the odds (LOD) of 3 and recombination frequency of 0.40. Genetic distance between markers in centimorgan (cM) unit was calculated using Kosambi’s mapping function [38].

### QTL analysis

QTLs controlling domestication-related traits were located onto the linkage map by inclusive composite interval mapping (ICIM) method [39] using the software QTL IciMapping 4.1 [40]. Probability value for entering variables in stepwise regression of phenotype on marker variables (PIN) was 0.001. ICIM was performed at every 1 cM. Significant log of odds (LOD) threshold for QTL analysis of each trait was determined by 3,000 permutation test at *P* = 0.05.

## Results

### SSR polymorphism

Out of 1,876 SSR primer pairs screened for polymorphism between the parents, 687 pairs (36.6%) were able to amplify DNA of the parents. Primers from azuki bean showed highest amplification rate (51.4%), while those from cowpea showed lowest amplification rate (19.2%). Among the 687 amplifiable primers, only 201 (29.3%) primers showed polymorphism between the parents. Percentage of polymorphic primers ranged from 20.4 (mungbean primers) to 32.7 (azuki bean primers) (S1 Table).

### RAD-seq markers

Illumina sequencing with HiSeq 2000 yielded a total of 29,568,103,697 RAD-tag 51-base reads from 578,901,731 raw reads. The number of RAD-tags of parents (AusTRCF66514 and JP235863) and F_2_ individuals was 3,124,320, 2,700,159 and 573,077,252, respectively. The average number of RAD-tags per F_2_ individual was 4,122,857. RAD-tags were aligned and clustered into 6,576 stacks. Average fill gaps rated for this 139 F_2_ individuals was 0.747 (max = 0.949, min = 0). Among these stacks, 479 RAD markers showed homozygote polymorphic genotype between parents. However, among these 479 RAD markers, 362 RAD markers were discarded because they had percentage of missing data more than 10%.

### Linkage map analysis

**Fig 2:**
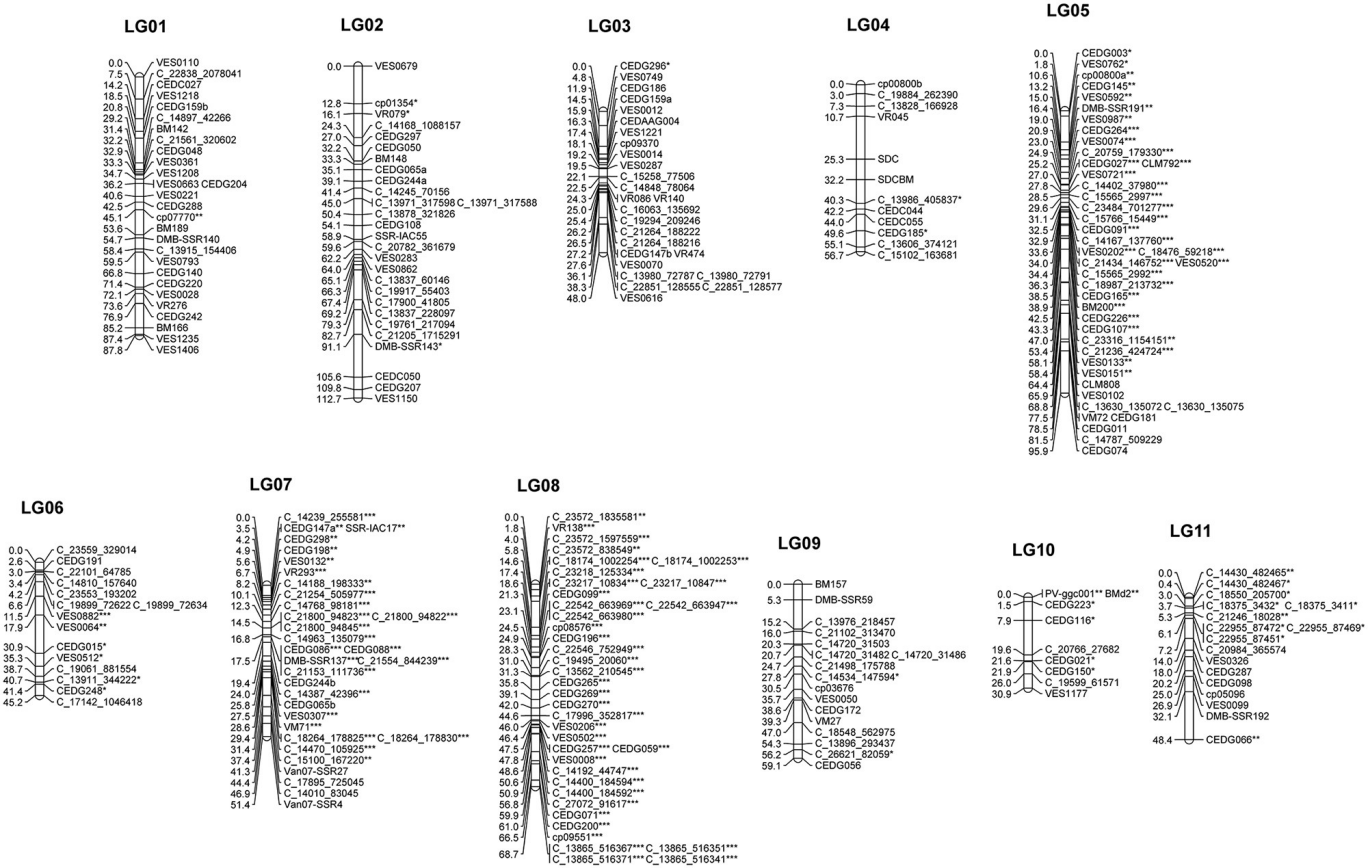
A genetic linkage map of *V*. *vexillata* developed from F_2_ population of 139 individuals from crossed between AusTRCF66514 and JP235863. Map distance and marker names are shown on the left and right side of the linkage groups, respectively. Markers showing significant deviation from the expected segregation ratio at 0.05, 0.01 and 0.001 probability levels are indicated with *, ** and ***, respectively. Marker names with prefixes C_ are RAD-Seq marker. SSR marker names with prefixes CED, Van07 and VES are from azuki bean. Marker names with prefixes VR and DMB-SSR are from mungbean. Marker names with prefixes cp and VM are from cowpea. Marker names with prefixes BM, BMd, SSR-IAC and PV are from common bean. SDC and SDCBM are morphological markers.

Finally, 264 markers (145 SSR, 117 RAD and 2 morphological markers) were used for the linkage map construction after discarding markers showing identical F_2_ genotypes, missing data more than 10%. Among these markers, as high as 128 (48.5%) showed segregation distortion. The 264 markers were clustered into 11 linkage groups (LGs 1–11) (Table 2 and Fig 2). The map spanned 704.8 cM in total, with a mean distance between markers of 2.87 cM. The lengths of linkage groups ranged from 30.9 cM (LG10) to 112.7 cM (LG2). A gap between markers more than 15 cM presented on only LG11 (between markers DMBSSR192 and CEDG066).

**Table 2:** Number of markers and average distance between markers in each linkage group of zombi pea F_2_ population (AusTRCF66514 x JP235863).

Linkage group (LG)	No. of SSR markers				No. of RAD-Seq markers	Total number of markers	Total length (cM)	Average distance between markers
	mungbean	azuki bean	cowpea	common bean				
**1**	2	18	1	3	4	28	87.8	3.14
**2**	2	11	1	2	12	28	112.7	4.03
**3**	3	12	1	0	10	26	48.0	1.85
**4**	1	3	1	0	5	14	56.7	4.05
**5**	1	21	4	1	15	42	95.9	2.28
**6**	0	6	0	0	9	15	45.2	3.01
**7**	2	11	1	1	17	32	51.4	1.61
**8**	1	12	2	0	23	38	68.7	1.81
**9**	1	3	2	1	10	17	59.1	3.48
**10**	0	5	0	2	2	9	30.9	3.43
**11**	1	5	1	0	10	17	48.4	2.85
**Average**	1.27	9.73	1.27	0.91	10.64	23.82	64.07	2.87
**Total**	14	107	14	10	117	262	704.8	31.54

**Note** SDC and SDCBM on LG4 are morphological markers

LGs 5, 6, 7, 8, 10 and 11 possessed the several/many distorted markers. The distorted markers clustered together. All the markers on LG8 were distorted markers. LGs 5 and 8 possessed many markers showing severe segregation distortion (*P*< 0.001). All the distorted markers on LG5 showed excessive cultivated parent genotype. Majority of the distorted markers on LGs 6 and 11 and about half of the distorted markers on LG10 showed excessive heterozygous genotype. All the distorted markers on LGs 7 and 8 and about half of the distorted markers on LG10 showed excessive homozygous wild genotype.

Primers CEDG065 and CEDG244 each amplified two different loci (markers). One of the loci from CEDG065 (named as CEDG065a) and one of the loci from CEDG244 (named as CEDG244a) were mapped near each other on LG2. The other loci from CEDG065 (named as CEDG065b) and the other loci from CEDG244 (named as CEDG244b) were mapped adjacent to each other on LG7.This indicated partial genome duplication and orthologous block of LG2 to LG7 or vice versa.

### Comparative linkage analysis

**Fig 3:**
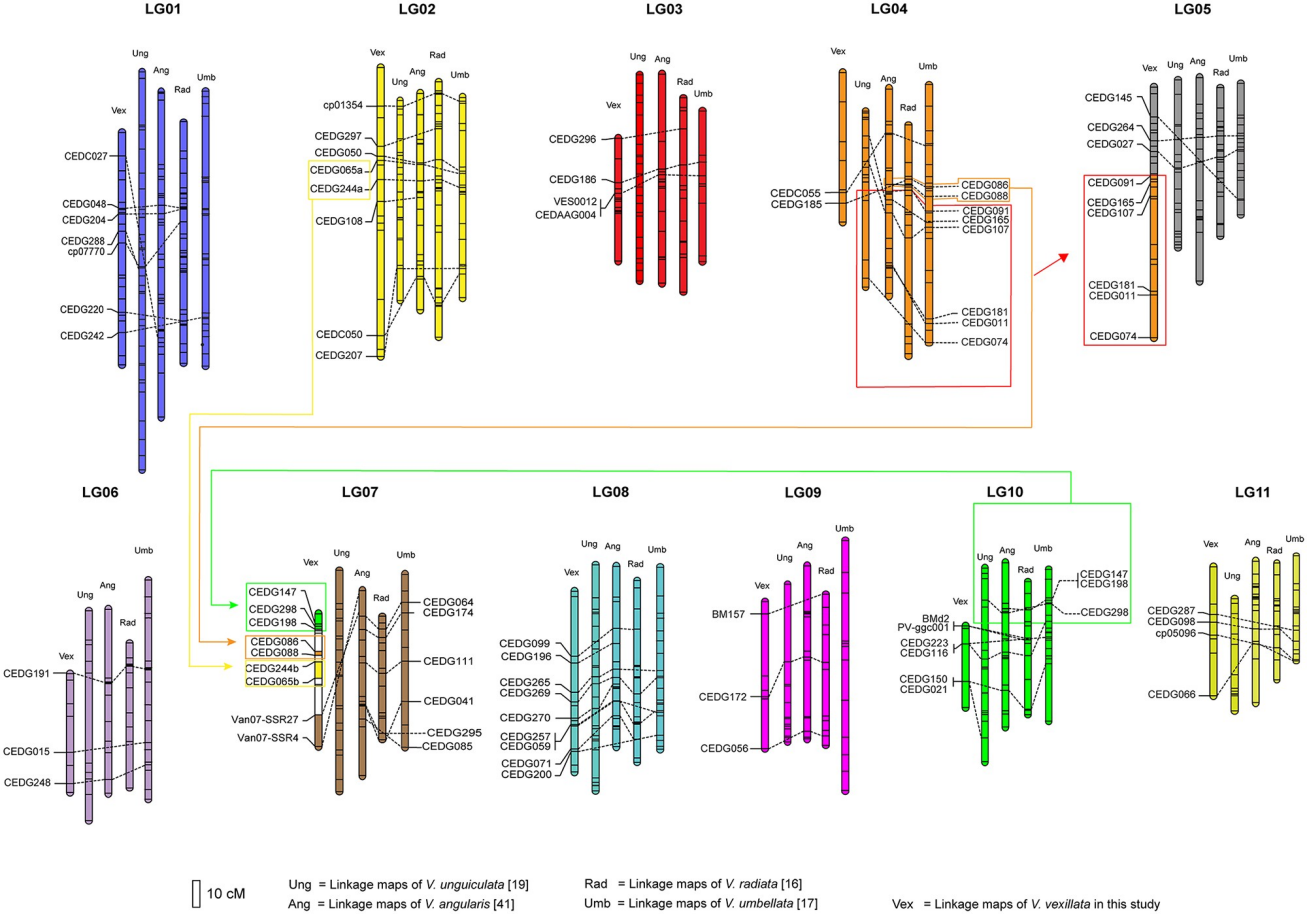
Comparative linkage map among *V*. *vexillata* (Vex), *V*. *unguiculata* (Ung), *V*. *angularis* (Ang), *V*. *radiata* (Rad) and *V*. *umbellata* (Umb) based on common SSR markers. Dotted lines connected common SSR markers between linkage maps.

The linkage map of zombi pea developed in this study were compared with previous linkage maps developed for *Vigna* crops including yardlong bean [19], azuki bean [41], mungbean [16] and rice bean [17] by using common SSR markers (Fig 3). The comparison revealed that in general the linkage groups and orders of the markers among the *Vigna* species were the same or highly similar. Nonetheless, remarkable macro genome rearrangements were found on LG5 and LG7 of our linkage map for *V*. *vexillata*. About a half of LG5 (middle to bottom) appeared to be an orthologous blocks from LG4, while a large portion of LG7 appeared to be orthologous blocks from LG4 and LG10. In addition, a small portion of the LG7 appeared to be duplication from LG2, and vice versa. The length of the zombi pea linkage map developed in this study was shorter that the linkage maps of others *Vigna* species.

### Variation of domestication-related traits

The parents were clearly different in all the traits recorded. The cultivated zombi pea showed higher value of trait value than the wild zombi pea in all the traits except pod shattering, seeds per pod, tubers per plant, and tuber length. For qualitative traits, the cultivated parent had yellow seed, while the wild zombi pea had brown seed with black mottle (Fig 1). The mean, range and standard deviation, and broad-sense heritability of the quantitative traits are shown in Table 3. Mean of the traits of the F_2_ population was between mean of parents for all traits except pod length, tuber length, 10^th^ node length and number of seeds per pod.

**Table 3:** Means, standard deviation (S.D.), minimum, maximum and heritability values for the parents and the F_2_ population that derived from a cross between wild (AusTRCF66514) and cultivated (JP235863).

Trait	Units	AusTRCF66514 (wild)				JP235863 (cultivated)				F_2_ population				Heritability (%)
		Mean	S.D.	Min	Max	Mean	S.D.	Min	Max	Mean	S.D.	Min	Max	
**PDT**	-	2.0	0.2	1.8	2.4	1.2	0.4	0.5	1.5	1.6	0.7	0.5	3.8	83.0
**SD100WT**	g	2.1	0.3	1.8	2.5	7.3	0.3	6.8	7.7	4.9	1.4	2.0	8.5	95.2
**SDL**	mm	3.9	0.3	3.6	4.4	6.4	0.2	6.2	6.7	5.7	0.5	4.7	6.8	79.6
**SDW**	mm	3.2	0.1	3.1	3.4	4.2	0.2	4.1	4.4	4.0	0.4	3.1	5.0	87.8
**SDT**	mm	2.3	0.2	1.9	2.7	3.9	0.2	3.7	4.2	3.1	0.4	2.2	4.2	63.3
**PDL**	mm	85.6	1.7	83.1	89.1	94.3	16.3	61.0	112.9	75.8	12.7	41.7	108.1	16.3
**PDW**	mm	3.6	0.1	3.5	3.7	5.7	0.2	5.2	5.9	5.1	0.4	4.2	6.0	80.3
**STT**	cm	2.6	0.3	2.3	3.0	3.9	0.4	3.1	4.6	3.8	0.6	2.4	5.8	69.2
**LFPL**	mm	29.1	2.6	24.9	34.1	54.8	6.7	43.1	63.0	49.4	7.8	28.2	71.2	58.0
**LFPW**	mm	10.0	0.8	8.6	11.8	27.9	2.7	21.2	30.3	20.9	4.7	12.8	34.7	81.8
**LFML**	mm	61.4	4.9	54.7	69.3	75.4	5.5	67.3	85.3	69.3	9.9	42.3	99.0	65.2
**LFMW**	mm	17.9	2.3	14.3	21.7	58.1	4.6	50.7	63.7	32.4	8.8	14.7	57.0	86.6
**NTB**	count	7.5	3.3	1.0	10.0	2.1	1.4	1.0	4.0	5.0	3.0	1.0	13.0	27.7
**TBW**	mm	15.8	1.7	13.2	18.5	39.4	6.6	29.0	47.3	22.6	7.3	9.3	40.0	56.3
**TBL**	mm	95.7	11.4	77.5	108.0	81.5	12.4	60.2	101.0	100.0	17.0	56.9	165.6	50.7
**TBWT**	g	17.8	6.5	9.1	27.8	60.7	11.9	46.9	84.1	46.0	30.2	5.8	143.0	89.9
**STL10**	cm	99.8	4.3	95.0	105.0	113.6	3.1	106.0	117.0	115.9	9.1	92.0	140.0	83.4
**FLD**	day	72.4	1.6	70.0	75.0	102.6	8.4	92.0	116.0	80.7	9.1	65.0	115.0	55.3
**PDDM**	day	27.8	1.4	26.0	30.0	34.0	1.4	32.0	36.0	32.8	3.2	16.0	39.0	80.5
**SDNPPD**	count	12.7	0.8	10.7	13.6	7.6	2.4	3.0	11.0	4.1	1.8	1.0	9.3	5.7
**SDC**	-	Brown				Yellow				Brown: Yellow				-
**SDCBM**	-	Present				Absent				Absent: Present				-

The measured traits in the F_2_ population showed nearly a normal distribution (Table 3). PDT, SD100WT, SDL, SDW, SDT, PDW, STT, LFPW, LFML, LFMW, TBWT, STL10 and PDDM showed moderate to high heritability (>60%), whereas LFPL, TBW, TBL and FLD showed medium to low heritability (<60%). PDL, NTB and SDNPPD showed heritability lower than 30%.

There was significant and positive correlation (*P*<0.05) between related traits in F_2_ population, such as between seed-related traits (SD100WT, SDL, SDW and SDT), between pod width and seed-related traits (SD100WT, SDL, SDW and SDT), between SDNPPD and PDL, between LFPW and LFMW, between TBW and TBWT (S2 Table). Negative correlation was found between TBW, TBWT and SDNPPD, STL10 and LFML and NTB, TBL and FLD (S2 Table). In general, correlation between related traits was moderate to high (>0.50), while correlation between non-related traits was low or none.

### QTLs for domestication-related traits

The results of the QTL analysis for each trait in F_2_ population are shown in Table 4. Out of 22 traits, ICIM did not find any QTL for four traits. Among those 18 traits which QTLs were identified, six traits each had only one QTL.

**Table 4:** Domestication-related QTLs detected in the F_2_ population from the cross between wild (AusTRCF66514) and cultivated (JP235863) *V*. *vexillata*.

Trait^a^	QTL name	LG^b^	Position^c^	Flanking markers	LOD	PVE^d^(%)	Add^e^	Dom^f^
**PDT**	Not detected							
**SD100WT**	*Sd100wt2*.*1-*	2	27	CEDG297 -CEDG050	5.38	12.78	-0.72	0.19
	*Sd100wt7*.*1-*	7	4	SSR-IAC17 -CEDG298	10.46	28.19	-1.04	0.17
**SDL**	*Sdl7*.*1-*	7	8	VR293 -C_14188_198333	7.95	32.60	-0.37	-0.03
**SDW**	*Sdw7*.*1-*	7	3	C_14239_255581 -CEDG147a	6.48	24.02	-0.24	0.02
**SDT**	*Sdt2*.*1-*	2	24	VR079 -C_14168_1088157	4.10	11.90	-0.18	0.01
	*Sdt5*.*1-*	5	25	C_20759_179330 -CEDG027	9.87	29.52	-0.29	0.16
	*Sdt8*.*1-*	8	44	CEDG270 -C_17996_352817	3.62	9.84	-0.26	-0.16
**PDL**	*Pdl4*.*1+*	4	27	SDC -SDCBM	3.84	7.66	2.66	8.53
	*Pdl4*.*2+*	4	48	CEDC055 -CEDG185	9.89	20.65	2.65	-14.36
	*Pdl5*.*1+*	5	24	VES0074 -C_20759_179330	4.97	9.32	1.86	-10.80
**PDW**	*Pdw1*.*1-*	1	73	VES0028-VR276	4.04	12.71	-0.19	-0.01
	*Pdw2*.*1-*	2	44	C_14245_70156 -C_13971_317598	5.70	18.11	-0.22	-0.09
	*Pdw7*.*1-*	7	3	C_14239_255581—CEDG147a	5.13	16.28	-0.21	0.03
**STT**	*Stt7*.*1-*	7	16	C_21800_94845—C_14963_135079	3.44	11.25	-0.30	-0.08
**LFPL**	*Lfpl8*.*1-*	8	47	VES0502—CEDG257	3.84	17.70	-1.73	3.69
**LFPW**	*Lfpw1*.*1-*	1	13	C_22838_2078041—CEDC027	3.65	5.90	-1.55	-0.52
	*Lfpw3*.*1-*	3	17	CEDAAG004—VES1221	11.01	19.58	-2.91	-0.56
	*Lfpw5*.*1-*	5	63	VES0151—CLM808	17.37	34.03	-3.47	-1.11
**LFML**	*Lfml5*.*1+*	5	62	VES0151—CLM808	5.49	17.19	0.53	0.14
**LFMW**	*Lfmw3*.*1-*	3	22	VES0287—C_15258_77506	9.88	12.72	-0.45	0.18
	*Lfmw5*.*1-*	5	61	VES0151—CLM808	29.37	52.16	-0.84	-0.37
**NTB**	Not detected							
**TBW**	*Tbw1*.*1-*	1	43	CEDG288—cp07770	4.89	11.14	-2.59	-1.55
	*Tbw2*.*1+*	2	67	C_19917_55403—C_17900_41805	3.97	9.02	2.64	-0.45
	*Tbw3*.*1-*	3	28	VES0070—C_13980_72787	3.61	8.30	-2.64	-0.58
	*Tbw4*.*1+*	4	3	cp00800b—C_19884_262390	4.00	8.88	1.00	3.60
	*Tbw8*.*1-*	8	67	cp09551—C_13865_516367	6.84	16.23	-3.53	1.00
**TBL**	Not detected							
**TBWT**	*Tbwt2*.*1+*	2	59	SSR-IAC55—C_20782_361679	4.91	9.78	11.90	5.83
	*Tbwt4*.*1+*	4	4	C_19884_262390—C_13828_166928	5.72	11.22	7.37	17.38
	*Tbwt8*.*1-*	8	57	C_27072_91617—CEDG071	6.98	14.66	-16.65	4.27
**STL10**	Not detected							
**FLD**	*Fld1*.*1-*	1	21	CEDG159b—C_14897_42266	5.72	17.37	-4.25	-4.17
**PDDM**	*Pddm2*.*1-*	2	69	C_17900_41805—C_13837_228097	4.72	18.92	-1.71	0.70
	*Pddm2*.*2+*	2	105	DMBSSR143—CEDC050	3.86	15.79	0.22	-2.36
**SDNPPD**	*Sdnppd 5*.*1-*	5	29	C_15565_2997—C_23484_701277	4.93	8.63	-0.10	-1.18
	*Sdnppd7*.*1+*	7	3	C_14239_255581—CEDG147a	11.59	25.14	0.04	-2.00
	*Sdnppd7*.*2-*	7	47	C_14010_83045—Van07-4	6.52	11.89	-0.25	1.33
	*Sdnppd8*.*1+*	8	46	C_17996_352817—VES0206	4.91	8.25	0.29	-0.94
	*Sdnppd9*.*1+*	9	25	C_21498_175788—C_14534_147594	3.89	6.37	0.26	0.92

^a^Trait abbreviation is described in Table 1,

^b^ Linkage group,

^c^ Position of QTL on LG,

^d^ Percentage of variance explained by the QTL,

^e^Additive effect and

^f^ Dominant effect

#### Pod dehiscence (PDT)

Both cultivated and wild parents showed small difference in number of twists along the pod (Table 3). In the F_2_ population, there was little variation of the trait. As a result, no QTL was detected for this trait.

#### Increase in organ size

Seed size, pod size, stem thickness, leaf size and tuber-related traits were considered.

#### Seed size (SD100WT, SDL, SDW and SDT)

One to three QTLs for traits related to seed size were located on LGs 2, 5, 7 and 8. At all QTLs detected, alleles from the cultivated parent increase the seed size. The QTLs with the largest phenotypic effects for all four traits were located on LG7 (28.19%, 32.60% and 24.02% for seed weight, seed length and seed width, respectively).

#### Pod size (PDL and PDW)

**Fig 4:**
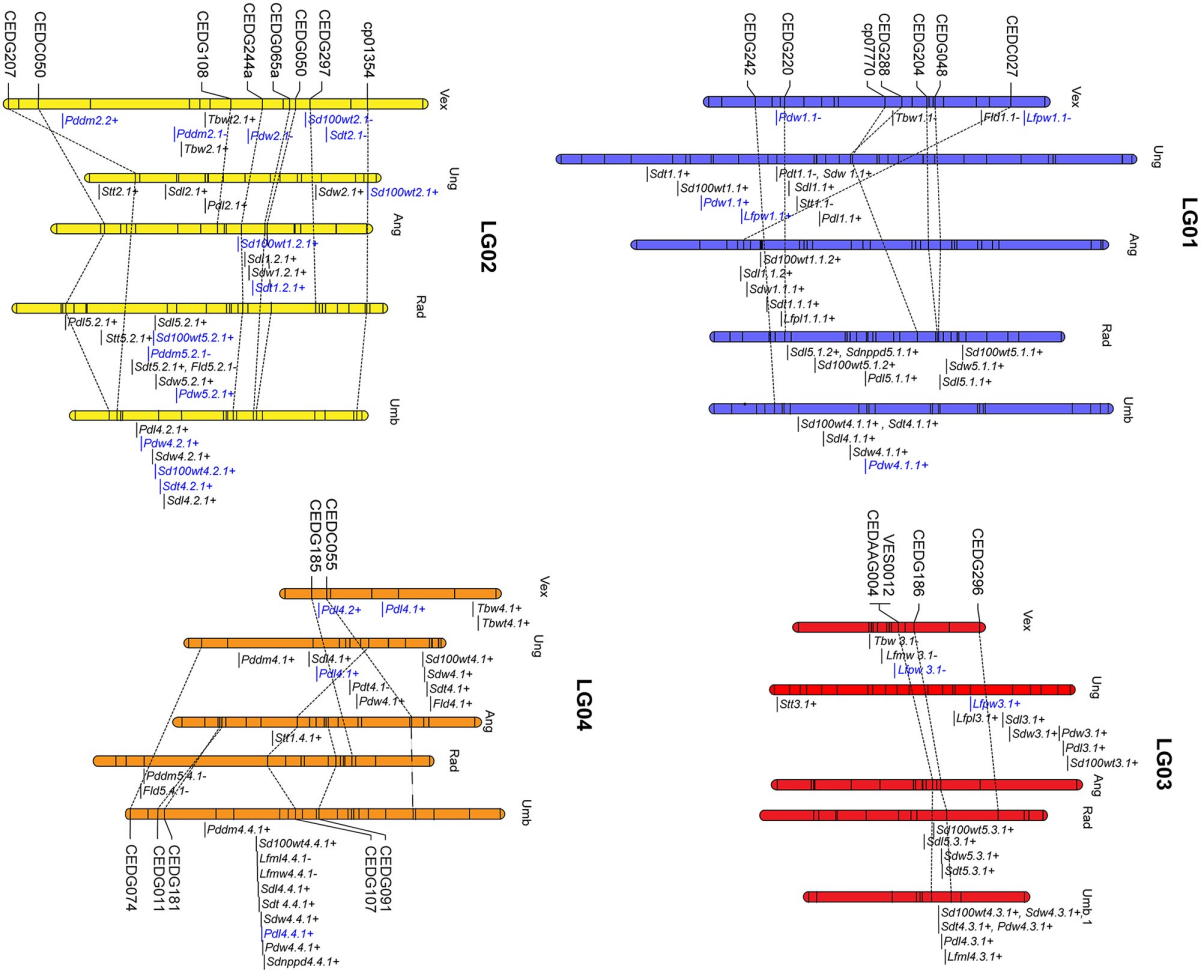
QTLs detected for domestication-related traits in *V*. *vexillata* F_2_ population of the cross between wild (AusTRCF66514) and cultivated (JP235863) accessions. The effect of the cultivated parent is indicated after each name. Explanation of trait abbreviation is shown in Table 1. QTL names in blue color are QTLs that are also identified on same linkage group(s) of other *Vigna* species. Bold QTLs names are common QTLs between/among species.

The cultivated zombi pea had larger pod size than the wild zombi pea. Three QTLs for each PDL and PDW traits were detected on LGs 1, 2, 4, 5 and 7. The QTLs with the highest contributions to these traits (PVE = 20.65% and 18.11%) were found on LGs 4 and 2. QTLs for both PDL and PDW on LGs 5 and 7 were located close to QTLs for seed size traits (Fig 4).

#### Stem thickness (STT)

Only one minor QTL (PVE = 11.25%) was detected on LG7 for stem thickness which was influenced by alleles from the cultivated parent that has thicker stem than the wild zombi pea.

#### Leaf size (LFPL, LFPW, LFML and LFMW)

Only one QTL was detected on LG8 for LFPL. Three QTLs for LFPW were detected on LGs 1, 3 and 5. One QTL was detected on LG5 for LFML. Two QTLs for LFMW were detected on LGs 3 and 5. The QTLs on LG5 for LFPW, LFML and LFMW showed highest PVE and were located very close to each other and likely to be the same locus. Similarly, the QTLs located on LG3 for LFPW and LFMW were located not far from each other. Alleles from the cultivated zombi pea increased leaf width, while alleles from the wild zombi pea increased leaf length.

#### Tuber traits (NTB, TBW, TBL and TBWT)

No QTL was detected for NTB and TBL. These two traits showed small trait variation and/or low heritability (27.7% for NTB) (Table 3). For TBW, five QTLs were identified on LGs 1, 2, 3, 4 and 8 in which the QTL on the LG8 (*Tbw8*.*1*-) had the largest effect (PVE = 16.23%, Table 4). Cultivated alleles on LGs 1, 3, 8 increased TBW. On the contrary, wild alleles on LG 2 (*Tbw2*.*1+*) and LG 4 (*Tbw4*.*1+*) increased TBW. In case of TBWT, three QTLs were detected on LGs 2, 4 and 8. Again, QTL on the LG8 (*Tbwt8*.*1-*) showed the highest PVE (14.66%). Cultivated allele on LG8 increased TBWT. On the contrary, wild alleles on LG 2 (*Tbwt2*.*1+*) and LG 4 (*Tbwt*4.1+) increased TBWT. QTLs for tuber width and tuber weight having phenotypically reverse effect (*Tbw2*.*1+* vs. *Tbwt2*.*1+*, and *Tbw4*.*1+* vs. *Tbwt4*.*1+*) were co-localized (Fig 4).

#### Plant type

Stem length was considered.

#### Stem length (STL10)

No QTL identified for this trait, although the trait showed high heritability (83.4%).

#### Earliness

Days to first flowering and days to first maturity were considered.

#### Days to first flowering (FLD)

The cultivated and the wild parents were very different in FLD (102.6 and 72.4 days, respectively). The F_2_ population showed a high variation for the trait (Table 3). However, only one QTL was detected for this trait. The QTL was on LG1 and accounted for only 17.37% of the trait variation. The alleles from the cultivated parent prolong FLD.

#### Days to first maturity (PDDM)

Similar to FLD, the cultivated and the wild parents were different in PDDM (34.0 and 27.8 days, respectively). Two QTLs on LG2, *Pddm2*.*1-* and *Pddm2*.*2+*, were identified for the trait. These QTLs showed similar PVE, 18.92% and 15.79%, respectively. The alleles from the cultivated parent at *Pddm2*.*1-* prolong PDDM, while that at *Pddm2*.*2+* hasten PDDM.

#### Yield potential

Or Number of seed per pod (SDDNPPD). The cultivated parent produced lower SDDNPPD than the wild parent. Five QTLs for SDDNPPD were detected LGs 5, 7, 8 and 9. Two QTLs on LG7 had high effects (PVE = 25.14% and 11.89%). At QTLs *Sddnppd5*.*1-* and *Sddnppd7*.*2-*, the allele from the cultivated parents increased SDDNPPD, while at the other three QTLs the alleles from the wild parent increased SDDNPPD.

#### Pigmentation

Seed coat color (SDC) and black mottle on seed coat (SDCBM)

#### Seed coat color (SDC)

SDC was characterized as a qualitative trait. The cultivated parent had yellow seed coat but wild parent had brown seed coat color. F_2_ plants segregated for this trait at ratio of 81 (brown) to 24 (yellow), fitting a 3:1 ratio (χ^2^ = 0.26, *P* = 0.61). This indicated that SDC is controlled by a single locus, named as *Sdc*. This locus was mapped as a morphological marker onto LG4 (Fig 2).

#### Black mottle on seed coat (SDCBM)

SDCBM was also characterized as a qualitative trait. The cultivated Bali had no black mottle, while the wild parent had black mottle on seed coat. F_2_ progenies segregated for seed coat color at ratio of 76 (black mottle presence) to 29 (black mottle absence), fitting a 3:1 ratio (χ^2^ = 0.38, *P* = 0.54)**.** This indicated that SDCBM is controlled by a single locus. The resulted indicated that the presence of SDCBM is controlled by a single dominant locus, named as *Sdcbm*. *Sdcbm* was mapped next to the locus *Sdc* at marker interval VR045 and C_13986_405837 (Fig 2).

### Distribution of domestication-related traits

QTLs with large effect (PVE > 20%) were found on four linkage groups, viz. LGs 4, 5 and 7 (Table 4).

#### LG4

Large-effect QTLs for pod length (*Pdl4*.*2+*) was on this linkage group. In addition, seed coat color (SDC) and black mottle (SDCBM) were mapped as morphological makers onto this linkage group.

#### LG5

Three large-effect QTLs were located to this linkage group; *Sdt5*.*1-* for seed thickness and *Lfpw5*.*1-* and *Lfmw5*.*1-* for leaf width. *Lfpw5*.*1-* and *Lfmw5*.*1-* with PVE = 34.03% and PVE = 52.16%, respectively were located closed together between markers VES0151 and CLM808. These two QTLs were likely the same locus. In addition, QTLs *Sdt5*.*1-*, *Pdl5*.*1+*, and *Sdnppd5*.*1-* were clustered together on this linkage group (Fig 4).

#### LG7

Most of large-effect QTLs for seed-related traits (*Sd100wt7*.*1-*, *Sdl7*.*1-* and *Sdw7*.*1-*) were located on this linkage group. These QTLs were clustered with QTLs for pod width (*Pdw7*.*1-*), number of seeds per pod (*Sdnppd7*.*1+*) and stem thickness (*Stt7*.*1-*). In addition, another QTL for number of seeds per pod (*Sdnppd7*.*2*-) was also located in this linkage group.

### Comparison of QTLs for domestication in *V*. *vexillata* with those of other *Vigna* species

The QTLs for domestication-related traits for zombi pea identified in this study were compared with those for yardlong bean [20], azuki bean [13], mungbean [16], and rice bean [17] based on common SSR markers (Fig 4). The comparison showed that most of the QTLs detected for zombi pea were also found in other *Vigna* species; they were mapped to the same linkage groups. For example, most of the QTLs for seed-related traits with high PVE in zombi pea were mapped onto LG7 in which several QTLs for seed-related traits were detected for yardlong bean, azuki bean, mungbean and rice bean. Three QTLs, *Pddm2*.*1-*, *Sdt8*.*1+* and *Sdnppd9*.*1-* were mapped to similar regions with other *Vigna* species. Nonetheless, none or a few of QTLs for seed-related traits were detected on LG6 and LG10 of zombi pea which is the same case as in other *Vigna* species except yardlong bean.

## Discussion

### Fertility of progenies of cultivated zombi pea from Bali x wild zombi pea

A previous study on reproductive compatibility among cultivated zombi pea from Bali (tuber type zombi pea),cultivated zombi pea from Africa (seed type zombi pea), and wild zombi pea from Africa and Australia revealed high compatibility between the African cultivated zombi pea (seed type zombi pea) and the wild form from both Africa and Australia, but revealed various pre- and post-zygotic incompatibility between the Bali cultivated zombi pea (tuber type zombi pea) and the wild form from Africa and Australia [8]. In our present study, we successfully obtained a fertile F_2_ population for the first time from a cross between the cultivated zombi pea from Bali and Indian wild zombi pea using the former as female parent. This suggested that the Bali cultivated zombi pea constitutes a primary gene pool with the Indian wild zombi pea. The difference between result in our study and that in Damayanti et al. [8] is possibly due to environmental factors. Damayanti et al. [8] noted that even self-pollination of the cultivated zombi pea from Bali results in low pod setting. In our study, when we conducted the hybridization during November 2014 to February 2015, the Bali cultivated zombi pea showed very low pod setting (<3%). When it was grown again during November 2015 to February 2016 and during November 2016 to February 2017, it set more flowers and pods (~15% and ~70%, respectively; Somta and Dachapak, personal observation). In addition to environmental factors, genetic relatedness of the parents appeared to affect the success of hybridization. The wild zombi pea (JP235863 from India) and the Bali cultivated zombi pea were genetically closely related [4], while the wild zombi pea used in the study of Damayanti et al. [8] were from Africa and Australia and they were genetically highly differentiated from the Bali cultivated zombi pea [4].

### Transferability and polymorphism of SSR markers

Although as high as 1,876 SSR primers pairs from various *Vigna* species were screened for polymorphism between the cultivated and wild parents, only 36.6% of them were amplifiable and only 10.7% of them were polymorphic. The low polymorphism of SSR markers was also observed in the mapping parents of *V*. *vexillata* used by Marubodee et al. [33] in which only 6.2% of 1,336 SSR markers were polymorphic. In addition, Dachapak et al. [4] reported that only 21.2% of 1,024 SSR markers screened in 6 accessions of *V*. *vexillata* from Asia, Africa, Australia and America were polymorphic. However, the percentage of amplifiable SSR markers in Marubodee et al. [33] and Dachapak et al. [4] (65.4% and 58.1%, respectively) was higher than in our study (36.6%). It is worth noting that almost all of the markers used by Marubodee et al. [33] and Dachapak et al. [4] were screened in our study. Nonetheless, these results indicate moderate transferability but low polymorphism of SSR markers from other *Vigna* species in *V*. *vexillata*. High transferability rate of SSRs (>80%) among several other *Vigna* species have been reported earlier [19,25,29,34,42]. The low transferability rate of SSRs from other *Vigna* species to *V*. *vexillata* is likely due to the fact that those *Vigna* species and *V*. *vexillata* are genetically highly differentiated [43]. Due to the low transferability and polymorphism of SSR markers in *V*. *vexillata*, other types of markers (RAD-seq in this study) should be used for genome analysis of *V*. *vexillata*.

### Linkage map construction and orthologous blocks in zombi pea

Previously, there was only two genetic linkage maps developed for zombi pea [33,44]. The first map contained 14 linkage groups with all were dominant markers (70 RAPD and 47 AFLP) except one co-dominant SSR marker. The map reported by Marubodee et al. [33] comprised 11 linkage groups from 84 SSR and 475 RAD-seq markers. The number of linkage map of zombi pea constructed in our study comprised 11 linkage groups of 145 SSR markers and 117 RAD-seq markers. The number of linkage groups of the maps developed by Marubodee et al. [33] and by this present study corresponded with the chromosome number of *V*. *vexillata*. (n = 11).

Previous comparative genome studies in the genus *Vigna* by means of comparison of linkage maps revealed high genome conservation among the species [17,19,25,42,45]. We compared our *V*. *vexillata* linkage map with other *Vigna* linkage maps using common SSR markers (Fig 3). We found that middle to lower part LG5 of our *V*. *vexillata* linkage map was very likely an orthologous block from LG4 and that LG7 of our *V*. *vexillata* linkage map was orthologous blocks from LG4 and LG10. In addition, a part of the LG7 appeared to be duplication of LG2 or vice versa (Fig 3). Intraspecific macro-orthologous block rearrangement has been demonstrated in azuki bean by means of comparative linkage mapping and fluorescence *in situ* hybridization [46,47]. The rearrangement of orthologous block was reciprocal where LG4 and LG6 were intermingled and was found in many accessions of wild azuki bean and it possibly affected fitness of those wild accessions to some environments and seed size [47].

### Segregation distortion

Segregation distortion is a normal phenomenon in interspecific hybridization and in even intraspecific hybridization between two highly genetically different genotypes such as between wild and cultivated form of the same species. Segregation distortion of DNA markers has been reported for intraspecific hybridization of several *Vigna* species including *V*. *radiata* [16], *V*. *angularis* [41,46], *V*. *umbellata* [17], *V*. *mungo* [42] and *V*. *unguiculata* [19,20]. The percentage of marker distortion in these reports ranged from 3.9% in *V*. *angularis* to 48.7% in *V*. *unguiculata*. In this study, as high as 48.5% of the markers showed segregation distortion, the value is considered very high as compared to that other reports in *Vigna* species. The distorted markers were clustered on LGs 5, 6, 7, 8, 10 and 11 (Fig 2). The presence of a gene(s) controlling sterility and/or compatibility may cause segregation distortion of nearby loci and clustering of distorted markers [48]. The highly distorted markers (*P*< 0.001) were on LGs 5, 7 and 8. The distortions found on LG5 and LG7 possibly stem from the chromosomal rearrangement in these two linkage groups (Fig 2; see also above discussion on orthologous block). The cause of the distortion on LG8 is not known, however, it is possible that this linkage group represents the most genome divergence between the wild and cultivated parents used in this study. It is worth noting that major QTLs for tuber-related traits (*Tbw8*.*1-* and *Tbwt8*.*1-*) were located on the LG8 (Fig 4 and Table 4). Tuber root trait in the cultivated *V*. *vexillata* from Bali used in this study is the most strikingly different adaptive/domestication trait distinguishing the cultivated *V*. *vexillata* Bali from other forms of *V*. *vexillata*. In yardlong bean (*V*. *unguiculata*), major QTLs for pod length and other pod-related traits (the most important domestication trait for this crop) and seed traits were found on LG7 where highly segregation distortions existed [19]. Similarly, in mungbean, major QTLs for seed dormancy, seed productivity and day length sensitivity were located on LG4 that showed high level of segregation distortion [16]. Most of markers mapped on LG11 of intra- and inter-specific crosses of *Vigna* species always showed segregation distortion [17,19,45]. This is also the case in our study where 58.8% of the markers mapped to LG11 showed segregation distortion (Fig 2). Therefore, LG11 appeared to play an important role in genetic differentiation within and among *Vigna* species.

Although pod length (PDL) and number of seed per pod (SDNPPD) were highly correlated (*r* = 0.83) (S2 Table), only one of the QTLs for these two traits were co-located (Table 4 and Fig 4). Since three of the five QTLs, including the largest effect QTL, detected for SDNPPD were mapped to genome regions that showed high segregation distortion, those QTLs may be associated with genetic compatibility and fertility, and thus seed production. In *Vigna* species, early generation (F_1_ and F_2_) hybrid progenies derived from hybridization of distantly-related genotypes always possesses pods with incomplete filled (empty seeds in some locules) [49,50]

### QTLs for tuber traits in zombi pea

Most of tubers crops are domesticated from non-legume species such as potato, sweet potato, yam and cassava. In *Vigna* species, *V*. *vexillata*, *V*. *lobatifolia* and *V*. *marina* produced tuber roots [51]. Tubers produced by legume crops have much more nutrition than non-legume crops especially nitrogen or protein due to nitrogen fixation ability. The protein content in tuber roots of *V*. *vexillata* is about 15% which is five-fold higher than that in sweet potato. In potato, tuber weight was controlled by a few QTLs with PVE of 20% or less [52]. Similarly, only three QTLs were identified for tuber weight (TBWT) in *V*. *vexillata* with largest PVE of about 15%. Tuber weight of potato has been shown to be negatively correlated with leaf area (leaf size) [53]. On the contrary, cultivated alleles that increase tuber width (*Tbw3*.*1-*), primary leaf width (*Lfpw3*.*1-*) and maximum leaflet width (*Lfmw3*.*1-*) were detected at similar position on LG3, which suggested preiotropic effect of the same gene (Table 4 and Fig 4). It should be noted that alleles from wild *V*. *vexillata* increased the tuber weight (*Tbwt2*.*1+*, *Tbwt4*.*1+*) and tuber width (*Tbw2*.*1*+, *Tbw4*.*1+*), in spite of the fact that wild *V*. *vexillata* has smaller tubers (Table 4 and Fig 1). Map positions of these QTLs are close and therefore tuber weight and tuber width increase by wild allele could be preiotropic effect of the same gene. These reverse effect alleles of wild *V*. *vexillata* may account for transgressive segregation found in the F_2_ population (S1 Fig). In fact, there is a F_2_ plant (F_2_-18) with 141–160 g range tuber weight, while tuber weight of cultivated parent is 61–80 g range (S1 Fig). Although QTL could not be detected, tuber number increase by wild Indian parent also contribute tuber weight increase. Therefore, these alleles from the wild *V*. *vexillata* will be useful for improvement of tuber yield of the Bali cultivated *V*. *vexillata*.

### Genomic region and distribution of QTLs for other domestication traits

Many domestication-related traits were studied in *Vigna* crops including mungbean [16], yardlong bean [19,20], azuki bean [13] and rice bean [17]. In general, these studies found that (i) domestication-related traits were controlled by one or two major QTLs together with some minor QTLs in a narrow genome region, (ii) major QTLs for different traits were clustered and (iii) QTL clusters were not randomly distributed along linkage groups. For examples, major QTLs for seed size, pod size, and seed dormancy were clustered on LG4 of mungbean, and major QTLs for pod length, seed size, pod shattering, leaf size, and stem thickness were clustered on LG7 of yardlong bean. In this study, we did not find clusters of major QTLs for different traits in zombi pea (Fig 4 and Table 4).

### Comparison of domestication QTLs between *V*. *vexillata* and yardlong bean

Since *V*. *vexillata* and is genetically closely related with cowpea/yardlong bean (*V*. *unguiculata*) and both of them originate from African, we compared domestication-related QTLs for *V*. *vexillata* found in this study with those for yardlong bean reported by [19,20]. Only QTLs having phenotypic effect more than 20% were considered (Table 5).

**Table 5:** Comparison of major QTLs (PVE > 20%) detected for domestication in F_2_ population (AusTRCF66514 x JP235863) of *Vigna vexillata* with the major QTLs (PVE > 20%) detected for domestication in other *Vigna* species.

Trait	Zombi pea		Yardlong bean		Azuki bean		Rice bean		Mungbean	
	QTL	PVE	QTL	PVE	QTL	PVE	QTL	PVE	QTL	PVE
**SD100WT**	*Sd100wt7*.*1 -*	28.2	*Sd100wt7*.*1+*	24.6	*Sd100wt2*.*1*.*1 +*	27.9	*Sd100wt4*.*4*.*1 +*	33.4	*Sd100wt5*.*8*.*1+*	22.2
					*Sd100wt1*.*2*.*1 +*	26.8				
					*Sd100wt2*.*2*.*1 +*	31.3				
					*Sd100wt2*.*9*.*1 +*	25.2				
**SDL**	*Sdl7*.*1 -*	32.6	*Sdl1*.*1 +*	20.8	*Sdl2*.*1*.*1+*	31.1	*Sdl4*.*4*.*1 +*	28.9	*Sdl5*.*8*.*1+*	20.4
			*Sdl7*.*1 +*	26.5	*Sdl1*.*2*.*1+*	24.5				
					*Sdl2*.*3*.*1+*	20.2				
**SDW**	*Sdw7*.*1 -*	24.0	*Sdw7*.*1+*	20.9	*Sdw1*.*2*.*1+*	21.2	*Sdw4*.*4*.*1 +*	26.3	dl	dl
					*Sdw2*.*9*.*1+*	21.2				
**SDT**	*Sdt5*.*1 -*	29.5	*Sdt3*.*1 +*	26.4	*Sdt1*.*2*.*1 +*	26.0	*Sdt4*.*4*.*1 +*	25.2	*Sdt5*.*8*.*1+*	22.7
					*Sdt2*.*9*.*1 +*	23.1				
**PDL**	*Pdl4*.*2 +*	20.7	*Pdl7*.*1 +*	31.0	*Pdl1*.*7*.*1+*	39.1	*Pdl4*.*4*.*1 +*	23.1	*Pdl5*.*7*.*1+*	20.5
					*Pdl1*.*8*.*1+*	22.1				
**LFPW**	*Lfpw5*.*1 -*	34.0	dl	dl	dl	dl	ni	ni	dl	dl
**LFMW**	*Lfmw5*.*1-*	52.2	ni	ni	dl	dl	*Lfmw4*.*4*.*1+*	25.6	ni	ni
**SDNPPD**	*Sdnppd7*.*1 +*	25.1	*Sdnppd11*.*1 +*	70.1	ni	ni	dl	dl	dl	dl

**Note** dl = detected but PVE lower than 20% and ni = not investigated

The cultivated zombi pea used in this study and yardlong bean are believed to have been domesticated in Asia, although their origin of species is believed to be in Africa [4,19]. Major QTLs of yardlong bean were detected on four linkage groups (LGs 1, 3, 7 and 11), while large-effect QTLs of zombi pea were found on three linkage groups (LGs 4, 5 and 7). Although some major QTLs were located on LG7 of both species, distributions of QTLs on this linkage group were different.

#### Seed weight

The 100-seed weight of the cultivated zombi pea was 7.3 g, while the wild zombi pea was only 2.1 g. Two QTLs for this trait were located on LG2 and LG7 (the largest effect (PVE = 28.19%) was on LG7). In yardlong bean, ten QTLs of 100-seed weight were detected on nine linkage groups with the highest QTL effect on LG7 (24.6%). Although the majors QTL for seed weight of zombi pea and yardlong bean were both located on the LG7, they appeared to be different because the QTL region in zombi pea was an orthologous block from LG10 as compared to yardlong bean (Fig 3).

#### Pod size

Pod size of the cultivated zombi pea is not much longer and thicker than that of the wild zombi pea (94.3 vs. 85.6 mm. for length and 5.7 vs. 3.6 mm. for width, respectively). Six QTLs for pod size in zombi pea was found on LGs 1, 2, 4, 5 and 7 with the largest effect QTL (PVE = 20.7%) on LG4. Seventeen QTLs for pod size in yardlong bean was found on every LGs except LG10 with the largest effect QTL on LG7 for both PDL and PDW (PVE = 31.0% and 31.2%, respectively). One of the QTL for pod size on LG4 might be shared between zombi pea and yardlong bean (Fig 4) was found between markers CEDC055 and CEDG185.

#### Leaf size

Both primary and mature leaves of the cultivated zombi pea were larger than the wild zombi pea. Seven QTLs were detected for leaf size with highest effect on LG5 (*Lfpw5*.*1-* with PVE = 34.03% and *Lfmw5*.*1-* with PVE = 52.16%). In yardlong bean, thirteen QTLs for primary leaf were detected on LG1, LG2, LG3, LG6, LG7, LG8, LG9 and LG11, but only one of them (*Lfpl7*.*1+*) with highest PVE (20.9%) was found on LG7.

#### Yield potential

Number of seeds per pod (SDNPPD) of the cultivated zombi pea was lower than that of the wild zombi pea (7.6 and 12.7, respectively). Five QTLs on LG5, LG7, LG8 and LG9 were detected with QTL showing highest PVE on LG7 (25.14%). In yardlong bean, two QTLs were detected on LGs 7 and 11. The QTL on LG11 showed highest PVE (70.1%).

### Comparison with azuki bean, mungbean and rice bean

Eight major QTLs (>20% PVE) of SD100WT, SDL, SDW, SDT, PDL, LFPW, LFMW and SDNPPD in zombi pea were compared with domestication-related QTLs from azuki bean [13], mungbean [16] and rice bean [17].

#### Seed size and weight

Seven QTLs of seed size and weight were detected for zombi pea in which QTLs with high effect were on LG7 (*Sd100wt7*.*1-*, *Sdl7*.*1-* and *Sdw7*.*1-*). In azuki bean, rice bean and mungbean, large-effect QTLs for seed size and weight were co-located on the same region; LGs 1, 2, 3 and 9 for azuki bean, LG4 for rice bean, and LG8 for mungbean. This suggested that major genes controlling seed size with different biological mechanisms exist among domesticated *Vigna* species.

#### Pod size

Among the QTLs for pod length and pod width in zombi pea, the QTL *Pdl4*.*2+* on LG4 showed the highest PVE (20.65%). QTL with the highest effect (PVE = 23.1%) for pod length in rice bean was also detected on LG4, while the QTL with the highest effect for pod length in azuki bean and mungbean were on LG7.

#### Leaf size

Among seven QTLs for leaf size in zombi pea, two QTLs (*Lfpw5*.*1-* and *Lfmw5*.*1-*) showed PVE higher than 20%; both of which were on LG5. In rice bean, a major QTL for leaf size was located on LG4. No QTL was detected for leaf size in azuki bean and mungbean due to small difference in the mapping parents.

#### Yield potential

Five QTLs for SDNPPD in zombi pea were located on LGs 5, 7, 8 and 9 with the QTL on LG7 showed highest PVE (25.14%), while three QTLs for SDNPPD in rice bean were detected on LG4 and LG9, and two QTLs for SDNPPD in mungbean were identified on LG1 and LG9. The QTLs on LG7 of zombi pea, rice bean, and mungbean were identified in a similar location and may be the same locus. However, all the QTLs identified in rice bean and mungbean possessed PVE lower than 20%.

#### Pigmentation

Seed coat color and presence of black mottle on seed coat were treated as morphological markers which were mapped next to each other on the LG4. In azuki bean and mungbean, gene controlling the presence of black mottle on seed coat was also mapped on LG4. This indicated that the gene controlling this trait is highly conserved among species in the genus *Vigna*.

## Conclusions

An F_2_ of *V*. *vexillata* population derived from a partially fertile hybrid between tuber-root-type cultivated form and wild type form were successfully developed. A genetic linkage map was constructed for this population utilizing 145 SSR, 117 RAD-seq and 2 morphological markers and to locate QTL for domestication syndrome traits. In total, 37 QTLs were detected for 18 domestication traits. Eight QTLs with large effect (>20%) were located on 4 out of 11 linkage groups. Domestication traits including seed size, pod size, leaf size, yield potential and seed pigmentation were controlled by one or two major QTLs and a few minor QTLs. QTLs for tubers were found on five different linkage group. Zombi pea had highest number of shared common QTLs with azuki bean, followed by yardlong bean, rice bean and mungbean. QTLs for seed size (SD100WT, SDL, SDW and SDT) were clustered together on different linkage groups of each species, suggesting specific and different seed size increase genes were used independently for each species. This study provides a genetic map and information for marker-assisted selection in zombi pea breeding. QTL analysis revealed potential of wild genetic resources to improve tuber size and yield. The comparative genomic analysis provides more understanding on genome evolution of *Vigna* species.

## Supporting information

S1 Fig Frequency distribution of 20 domestication related traits (except SDC and SDCBM traits) in F_2_ population of *Vigna vexillata* (AusTRCF66514 x JP235863). (TIFF)

S1 Table Summary of percentage of amplification and polymorphic markers from four *Vigna* species in *Vigna vexillata* accessions AusTRCF66514 and JP235863. (XLSX)

S2 Table Correlation between domestication traits in F_2_ population of *V*. *vexillata* (AusTRCF66514 x JP235863). (XLSX)
